# Intracellular Ca^2 +^ Imbalance Critically Contributes to Paraptosis

**DOI:** 10.3389/fcell.2020.607844

**Published:** 2021-01-12

**Authors:** Eunhee Kim, Dong Min Lee, Min Ji Seo, Hong Jae Lee, Kyeong Sook Choi

**Affiliations:** ^1^Department of Biological Sciences, Ulsan National Institute Science and Technology, Ulsan, South Korea; ^2^Department of Biochemistry, Ajou University School of Medicine, Suwon, South Korea

**Keywords:** paraptosis, Ca^2+^, endoplasmic reticulum, mitochondria, cancer

## Abstract

Paraptosis is a type of programmed cell death that is characterized by dilation of the endoplasmic reticulum (ER) and/or mitochondria. Since paraptosis is morphologically and biochemically different from apoptosis, understanding its regulatory mechanisms may provide a novel therapeutic strategy in malignant cancer cells that have proven resistant to conventional pro-apoptotic treatments. Relatively little is known about the molecular basis of paraptosis, but perturbations of cellular proteostasis and ion homeostasis appear to critically contribute to the process. Ca^2+^ transport has been shown to be important in the paraptosis induced by several natural products, metal complexes, and co-treatment with proteasome inhibitors and certain Ca^2+^-modulating agents. In particular, the Ca^2+^-mediated communication between the ER and mitochondria plays a crucial role in paraptosis. Mitochondrial Ca^2+^ overload from the intracellular Ca^2+^-flux system located at the ER–mitochondrial axis can induce mitochondrial dilation during paraptosis, while the accumulation of misfolded proteins within the ER lumen is believed to exert an osmotic force and draw water from the cytoplasm to distend the ER lumen. In this process, Ca^2+^ release from the ER also critically contributes to aggravating ER stress and ER dilation. This review focuses on the role of Ca^2+^ transport in paraptosis by summarizing the recent findings related to the actions of Ca^2+^-modulating paraptosis-inducing agents and discussing the potential cancer therapeutic strategies that may effectively induce paraptosis *via* Ca^2+^ signaling.

## Introduction

The term “paraptosis” was first introduced to describe a form of programmed cell death displaying cytoplasmic vacuolation consisting in mitochondrial and/or endoplasmic reticulum (ER) dilation (Sperandio et al., 2000). Paraptosis was initially observed in 293T cells and mouse embryonic fibroblasts overexpressing insulin-like growth factor 1 receptor (IGF-1R) (Sperandio et al., 2000) and was subsequently shown to be induced by different natural compounds *in vitro* and *in vivo* in tumor cells (Lee et al., 2016; Fontana et al., 2020b). In addition, paraptosis appears to be implicated in neurodegeneration (Wei et al., 2015). Paraptosis lacks apoptotic features (e.g., DNA condensation and fragmentation, membrane blebbing, and apoptotic bodies), and unlike necrosis, no loss of membrane integrity is seen in paraptosis (Sperandio et al., 2000, 2004). As the underlying mechanisms of paraptosis, impaired proteostasis (due to proteasomal inhibition or disrupted protein thiol homeostasis), and/or imbalanced homeostasis of ions (e.g., Ca^2+^ and K^+^) have been proposed to trigger stress to the ER and mitochondria (Lee et al., 2016; Fontana et al., 2020b). Paraptosis is successfully inhibited by the translation inhibitor, cycloheximide (CHX) (Sperandio et al., 2000), suggesting that inhibition of protein synthesis may prevent this cell death by reducing the potential burden of misfolded proteins and proteotoxicity (Lee et al., 2016). The activation of mitogen-activated protein (MAP) kinases, such as c-Jun N-terminal protein kinases (JNKs), MAP kinase kinase 2 (MEK-2), and p38, has been found to be positively involved in the paraptosis induced by many natural products or chemicals (Zhang et al., 2009, 2010; Yoon et al., 2010, 2012, 2014; Wang et al., 2012; Yumnam et al., 2014; Hager et al., 2018; Han et al., 2018; Fontana et al., 2019; Yokoi et al., 2020), whereas AIP-1/Alix appears to be negatively involved in some cases of paraptosis (Sperandio et al., 2004; Valamanesh et al., 2007; Yoon et al., 2010, 2014; Han et al., 2018; Xue et al., 2018). The term “paraptosis-like cell death” has been used to describe the types of cell death accompanied by the dilation of either the mitochondria or the ER alone or those that do not share multiple biochemical features of paraptosis, including the activation of MAP kinases, the downregulation of AIP-1/Alix, and the inhibition of vacuolation and cell death by cycloheximide, etc. (Zhang et al., 2010; Xue et al., 2018; Kim et al., 2019). Recently, Ca^2+^ depletion in the ER and the subsequent mitochondrial Ca^2+^ overload have been shown to play a critical role in the paraptosis induced by various natural products and chemicals (Yoon et al., 2012, 2014; Yumnam et al., 2016; Kim et al., 2019). Here we review the recent understanding of paraptosis, focusing on the involvement of intracellular Ca^2+^ transport in paraptosis, provide mechanistic insight into the Ca^2+^-mediated regulatory pathways of paraptosis, and finally discuss the cancer therapeutic strategy of inducing paraptosis by manipulating intracellular Ca^2+^ homeostasis.

## The Machinery Involved in the ER-Mitochondria Ca^2+^ Transport

The ER and mitochondria both participate in regulating intracellular Ca^2+^ homeostasis due to their ability to store Ca^2+^ and respond to cytosolic Ca^2+^ signals. Under normal physiological conditions, the concentrations of both cytosolic Ca^2+^ and Ca^2+^ stored within the ER lumen are strictly regulated. The cytosolic Ca^2+^ concentration [(Ca^2+^)c] is approximately 0.1 μM, compared to an extracellular (Ca^2+^) of ∼1 mM and an ER Ca^2+^ concentration [(Ca^2+^)er] of ∼0.5 mM (Bianchi et al., 2004). The import of Ca^2+^ into the ER is governed by sarcoplasmic/ER Ca^2+^ ATPase (SERCA) pumps (Vandecaetsbeek et al., 2011). In response to cellular stress requiring Ca^2+^-signal, Ca^2+^ is released from ER stores *via* ryanodine receptors (RyRs) and (especially) inositol 1,4,5,-triphosphate receptors (IP_3_Rs) (Marks, 1997); the latter are primarily clustered in the mitochondria-associated membrane (MAM), an ER structure that is located near the mitochondria (Rizzuto et al., 1998; Csordás et al., 1999; Bartok et al., 2019). The mitochondria serve as important regulators of cellular Ca^2+^ by sequestering and releasing Ca^2+^. The Ca^2+^ concentration (Ca^2+^) inside the mitochondria has similar values measured in the bulk cytoplasm (0.1–0.2 μM) under resting conditions; however, mitochondrial Ca^2+^ concentration increased 10—20-fold more than the cytosolic compartment during stimulation with (Ca^2+^)-increasing agents (Giorgi et al., 2018). In particular, Ca^2+^ ions released from the ER by IP_3_Rs or RyRs flux across the outer mitochondrial membrane (OMM) mainly through the voltage-dependent anion channel (VDAC) (Gincel et al., 2001; Rapizzi et al., 2002). After reaching the intermembrane space, Ca^2+^ ions pass through the inner mitochondrial membrane (IMM) mainly through the mitochondrial Ca^2+^ uniporter (MCU) complex (Baughman et al., 2011; De Stefani et al., 2011). This MCU complex consists of two pore-forming proteins; mitochondrial calcium uptake protein (MCU)1–3 (Baughman et al., 2011; De Stefani et al., 2011) and essential MCU regulator (Sancak et al., 2013); the dominant-negative pore-forming subunit, MCUb (Raffaello et al., 2013), and the scaffolding factor, mitochondrial calcium uniporter regulator 1 (MCUR1) (Mallilankaraman et al., 2012; Tomar et al., 2016). The activity of the MCU complex is regulated by mitochondrial calcium uptake 1 (MICU1) (Perocchi et al., 2010) and its paralog MICU2 (Plovanich et al., 2013); they together comprise the MCU complex, which allows for mitochondrial Ca^2+^ uptake exclusively at high Ca^2+^ concentrations (Patron et al., 2014). Therefore, the MCU complex has been proposed to be the key player responsible for the rate-limiting step of mitochondrial Ca^2+^ accumulation, and it may be pivotal to Ca^2+^-overload-induced cell death. The Ca^2+^ taken up by the mitochondria is rapidly extruded into the cytosol *via* a complex antiporter system to restore the basal state. The mitochondrial Na^+^/Ca^2+^ exchanger (mNCX) and the mitochondrial H^+^/Ca^2+^ exchanger (mHCX) play major roles in mitochondrial Ca^2+^ efflux mechanisms. The stoichiometry of mNCX-driven transport is electrogenic, with three (or four) Na^+^ for one Ca^2+^ (Jung et al., 1995; Dash and Beard, 2008), whereas the exchange ratio of mHCX is electroneutral (two H^+^ for one Ca^2+^) (Gunter et al., 1991). The mitochondrial permeability transition pore (mPTP) has been also proposed to be involved as an alternative Ca^2+^ efflux pathway under certain conditions when the mPTP is transiently opened (Elrod et al., 2010; Lu et al., 2016).

## Paraptosis-Inducing Agents Associated With Ca^2+^ Imbalance

**FIGURE 1:**
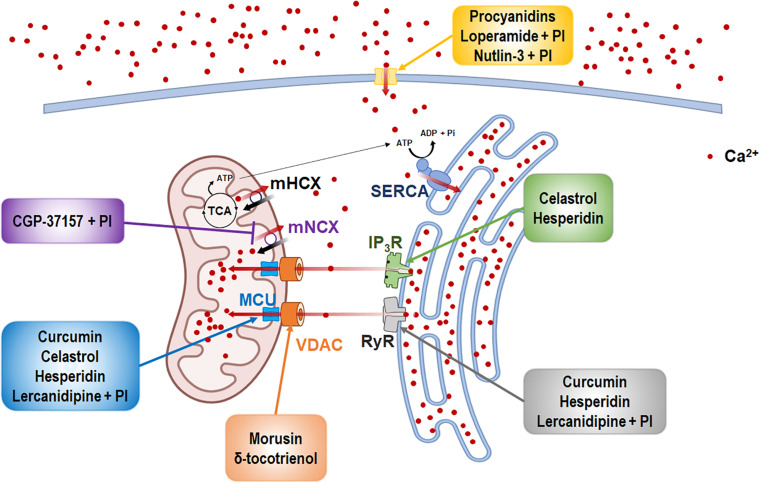
The inducers of Ca^2+^-mediated paraptosis and their molecular targets. Paraptosis is induced by drugs targeting the activities of several Ca^2+^ channels and transporters. The names of paraptotic inducers are given in colored boxes, and their potential targets are marked by arrows of corresponding colors. The figure was produced using Biorender (biorender.com).

The natural products and chemicals that are listed in Table 1 induce paraptosis. During this process, they disrupt Ca^2+^ homeostasis at the ER by altering Ca^2+^ store content and Ca^2+^ dynamics (including the uptake, release, and leakage of Ca^2+^) and/or at the mitochondria by affecting the activities of MCU complex components to regulate the mitochondrial Ca^2+^ levels. In addition, recent studies have shown that treatment of cancer cells with drugs that perturb Ca^2+^ homeostasis [CGP-37157 (Yoon et al., 2012), lercanidipine (Lee et al., 2019), and loperamide (Kim et al., 2019)] or Nutlin-3, which is a chemical that triggers the mitochondrial unfolded protein response (mtUPR) (Lee et al., 2017), induces paraptosis when combined with proteasome inhibitors (PIs). The proposed targets through which these paraptosis-inducing agents disrupt Ca^2+^ homeostasis are summarized in Figure 1. In this section, we will summarize and discuss their Ca^2+^-associated regulatory effects in paraptosis.

**TABLE 1:** Paraptotic inducers and their pro-paraptotic mechanisms of action.

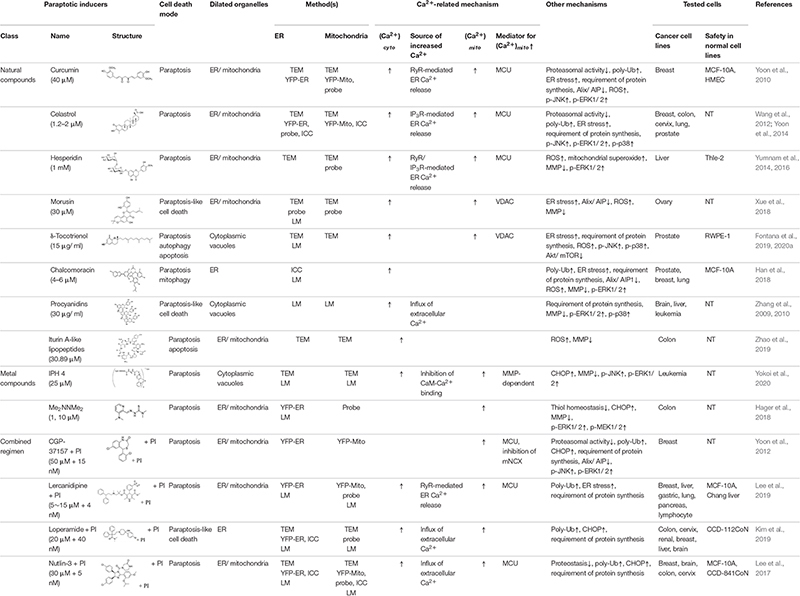

**Normal cell line: MCF-10A, human mammary epithelial cell line; HMEC, primary mammary epithelial cells; Thle-2, primary normal liver cells by infection with SV40 large T antigen; RWPE-1, human prostatic epithelial cells; IMR90, human lung fibroblast cells; Chang Liver, normal liver cell line; CCD-112CoN, human colon fibroblast cells; CCD-841CoN: human colon epithelial cells. TEM, transmission electron microscopy; LM, light microscopy; ICC, immunocytochemistry; ↑, upregulation; ↓, downregulation; (Ca^2+^)_*cy*__*to*_, concentration of cytosolic Ca^2+^; (Ca^2+^)_*mi*__*to*_, concentration of mitochondrial Ca^2+^.**

### Curcumin

Curcumin, the main natural polyphenol extracted from the rhizomes of *Curcuma longa*, demonstrates chemopreventive (Park et al., 2013), chemosensitizing, and radiosensitizing activity in cancer cells (Goel and Aggarwal, 2010). Yoon et al. (2010) showed that curcumin can induce paraptosis by promoting vacuolization *via* the swelling and fusion of the mitochondria and the ER in various breast cancer cell lines, but not in normal breast cells. The curcumin-induced vacuolation and cell death were effectively abrogated by the protein synthesis blocker, cycloheximide. The authors found that impairment of proteasome activity by curcumin is the main cause of paraptosis-related ER stress and ER dilation and that mitochondrial Ca^2+^ overload is needed for mitochondrial dilation seen during curcumin-induced paraptosis. More specifically, curcumin-induced activation of RyRs mediates the release of Ca^2+^ from the ER. Subsequently, MCU-mediated mitochondrial Ca^2+^ uptake causes Ca^2+^ overload, contributing to the dilations of the mitochondria and the ER, and cell death (Yoon et al., 2010). These results suggest that mitochondrial Ca^2+^ overload is an initial and important signal for curcumin-induced paraptosis. The activations of ERK2 or JNKs were also found to critically contribute to curcumin-induced paraptosis. Furthermore, the protein levels of AIP-1/Alix were decreased by curcumin, and AIP-1/Alix overexpression attenuated curcumin-induced paraptosis (Yoon et al., 2010).

### Celastrol

Celastrol, a quinone methide triterpene derived from Thunder God Vine, exhibits antioxidant, antidiabetic, antiobesity, and anti-tumor activity (Cascão et al., 2017). Celastrol has been found to trigger paraptosis in various cancer cells (Yoon et al., 2014). In HeLa cells, celastrol-induced paraptosis was found to be accompanied by apoptosis and autophagy, suggesting that different cellular fates could be induced by celastrol depending on the cell type and/or cellular context (Wang et al., 2012). Celastrol treatment of MDA-MB-435S cells was shown to induce mitochondrial Ca^2+^ overload and ER stress. IP_3_R-mediated Ca^2+^ release from the ER and its subsequent MCU-mediated mitochondrial Ca^2+^ influx appear to be critically implicated in celastrol-induced paraptosis. Mitochondrial Ca^2+^ overload results in its functional defect and the generation of reactive oxygen species (ROS), which further impair proteasome activity. This proteasome-inhibiting activity of celastrol may stabilize IP_3_R and MCU to reinforce the Ca^2+^-mediated effects of celastrol, leading to ER stress, ER vacuolation, and subsequent cell death (Yoon et al., 2014).

### Hesperidin

Hesperidin, a flavanone glycoside present in citrus fruits, was shown to kill HepG2 cells through the induction of paraptosis accompanied by mitochondrial and ER swelling without apoptotic features (Yumnam et al., 2014, 2016). Treatment with hesperidin was found to increase the mitochondrial Ca^2+^ levels in these cells, and MCU-mediated mitochondrial Ca^2+^ overload was revealed to play an important role in hesperidin-induced paraptosis. Ca^2+^-mediated increase in ROS generation was shown to induce the hesperidin-induced loss of mitochondrial membrane potential, and ERK1/2 activation was found to positively influence hesperidin-induced vacuolation (Yumnam et al., 2014, 2016). Both IP_3_R and RyR mediate the release of Ca^2+^ from the ER and subsequent MCU-mediated Ca^2+^ influx into the mitochondria, thereby contributing to the paraptosis induced by hesperidin in HepG2 cells (Yumnam et al., 2016).

### Morusin

Morusin, a prenylated flavonoid isolated from the root bark of *Morus australis*, exhibits anti-oxidant, anti-bacterial, and anti-tumor properties (Zoofishan et al., 2018). Treatment of epithelial ovarian cancer (EOC) cells with morusin triggers paraptosis-like cell death accompanied by extensive cytoplasmic vacuolation due to the dilation and fusion of the ER and the mitochondria (Xue et al., 2018). Morusin induced mitochondrial Ca^2+^ overload, accumulation of ER stress marker proteins, ROS generation, and loss of the mitochondrial membrane potential (MMP, ΔΨm) in these cells. These morusin-induced mitochondrial Ca^2+^ influx, ROS generation, MMP depletion, cytoplasmic vacuolization, and cell death were suppressed by a pretreatment with 4,4′-diisothiocyanostilbene-2,2′-disulfonic acid (DIDS), an inhibitor of anion channels, including VDAC (Jessen et al., 1986; Ben-Hail and Shoshan-Barmatz, 2016). In addition, DIDS inhibited the morusin-induced antiproliferative effects, which were associated with ER stress, both *in vitro* and *in vivo* in ovarian cancer xenograft models. In conclusion, morusin may have antitumor potential against EOC by inducing paraptosis *via* VDAC-mediated mitochondrial Ca^2+^ overload (Xue et al., 2018).

### δ-Tocotrienol

δ-Tocotrienol (d-TT) induces paraptosis together with autophagy and apoptosis in castration resistance prostate cancer (CRPC) cells. d-TT-induced paraptosis was demonstrated by the reported dilation of ER cisternae and swollen and damaged mitochondria with disintegrated cristae (Fontana et al., 2019). δ-TT-induced vacuolation was prevented by salubrinal (the ER stress inhibitor) and cycloheximide (the protein synthesis inhibitor). δ-TT was found to trigger significant impairments of mitochondrial metabolic functions and structural dynamics (Fontana et al., 2020a). In addition, δ-TT significantly increases the levels of cytoplasmic Ca^2+^, mitochondrial Ca^2+^, and ROS, which contribute to the pro-death activities (paraptosis, apoptosis, and autophagy) of this agent in these cells. Pretreatment with the VDAC inhibitor, DIDS, significantly attenuated the effects of δ-TT on cell viability (paraptosis and apoptosis), suggesting that mitochondrial Ca^2+^ overload is critical for the anticancer activity of δ-TT in CRPC cells (Fontana et al., 2020a).

### Chalcomoracin

Treatment with chalcomoracin (CMR), one of the major secondary metabolites found in fungus-infected mulberry leaves, induces mitophagy and a paraptosis that is accompanied by extensive cytoplasmic vacuolation originated from the ER in PC-3 and MDA-MB-231 cells (Han et al., 2018). In this process, CMR treatment upregulated PTEN-induced kinase 1, a key marker of mitophagy, and downregulated the protein levels of AIP-1/Alix, an inhibitory protein of paraptosis. CMR-induced ROS generation and MMP loss were effectively inhibited by a pretreatment with the antioxidant, N-acetylcysteine (NAC). Moreover, increases in the level of Ca^2+^ and the activation of the Ca^2+^-activated protease, calpain, preceded CMR-induced cytoplasmic vacuolation, and a pretreatment with the intracellular Ca^2+^ chelator, BAPTA-AM, blocked CMR-induced cytoplasmic vacuolation. These findings together suggest that ROS-mediated impairment of MMP and Ca^2+^ homeostasis may contribute to CMR-induced paraptotic cell death (Han et al., 2018).

### Procyanidins

Oligomeric procyanidins (F2), which are abundant in grape seeds, can induce paraptosis in U-87 cells (Zhang et al., 2009). Apoptotic features were not observed in cells treated with F2. However, a further study indicated that activation of the ERK1/2 and p38 pathways, as well as Ca^2+^ mobilization, was involved in the F2-mediated cell death of U-87 cell (Zhang et al., 2010).

### Iturin A-Like Lipopeptides

Iturin A-like lipopeptides produced by *Bacillus subtillis* were reported to simultaneously induce paraptosis, apoptosis, and autophagy in heterogeneous human epithelial colorectal adenocarcinoma (Caco-2) cells (Zhao et al., 2019). Induction of paraptosis in Caco-2 cells was shown by cytoplasmic vacuolization accompanied by ER dilation and swelling as well as mitochondrial dysfunction. The antitumor effect of the iturin A-like lipopeptides on Caco-2 cells was decreased by more than 50% in the presence of an apoptosis inhibitor, suggesting that paraptosis may play a crucial role in its anticancer activity. A significant increase in Ca^2+^ and ROS levels was also observed with ER stress in cells (Zhao et al., 2019).

### Triscyclometalated Iridium(III) Complex-Peptide Hybrids 4

Yokoi et al. (2020) demonstrated that the amphiphilic iridium complex-peptide hybrid (IPH) 4, which contains a glycolic acid moiety between the iridium core and the peptide part, induces paraptosis-like cell death in Jurkat cells (Yokoi et al., 2020). The authors found that IPH4 increased the influx of Ca^2+^ from the ER to the mitochondria and induced the subsequent loss of MMP (ΔΨm), resulting in vacuolization of the cytoplasm and intracellular organelles (Yokoi et al., 2020).

### Me_2_NNMe_2_

Me_2_NNMe_2_, which is a new derivative of α-N-heterocyclic thiosemicarbazones, induces major hallmarks of paraptotic cell death, including ER-derived vacuolation, mitochondrial swelling, activation of MEK/ERK signaling pathway, and caspase-independent cell death (Hager et al., 2018). In this process, the copper complex of Me_2_NNMe_2_ accumulates in the ER to inhibit the reductive potential of protein disulfide isomerase (PDI). The resultant disruption of thiol redox homeostasis in the ER, in turn, activates protein kinase R (PKR)-like endoplasmic reticulum kinase signaling and the release of Ca^2+^ ions from the ER. This prolonged Ca^2+^ imbalance triggers the swelling of the ER and the mitochondria as well as mitochondrial membrane depolarization, leading to cell death (Hager et al., 2018).

### CGP-37157 + Proteasome Inhibitor

Yoon et al. (2012) showed that proteasome inhibition alone is not effective to kill breast cancer cells. However, the CGP-37157-mediated inhibition of mNCX could sensitize breast cancer cells (but not normal cells) to a PI by inducing paraptosis accompanied by dilation of both the mitochondria and the ER (Yoon et al., 2012). In addition, co-treatment of CGP-37157 enhanced bortezomib (Btz)-mediated ER stress, ERK activation, JNK activation, and AIP-1/Alix downregulation. While the mitochondrial Ca^2+^ levels were transiently increased by CGP-37157 alone, CGP-37157/Btz induced a sustained mitochondrial Ca^2+^ overload. A pretreatment with ruthenium red significantly inhibited the cell death induced by CGP-37157/Btz, possibly by inhibiting the influx of Ca^2+^ into the mitochondria. These findings together indicate that the simultaneous inhibition of the proteasome and mNCX effectively induces paraptosis *via* mitochondrial Ca^2+^ overload and that dilations of the ER and the mitochondria seem to be interdependent during paraptosis (Yoon et al., 2012).

### Lercanidipine + Proteasome Inhibitor

A recent study showed that lercanidipine (Ler), a third-generation 1,4-dihydropyridine (DHP) used to treat high blood pressure, potentiates the antitumor effects of various PIs (e.g., Btz, carfilzomib, and ixazomib) in many solid cancer cell lines by inducing paraptosis, a cell death mode accompanied by extensive vacuolation derived from the ER and the mitochondria (Lee et al., 2019). Ler enhances Btz-mediated ER stress and ER dilation in MDA-MB 435S cells. Mitochondrial Ca^2+^ overload was followed by the increase in cytosolic Ca^2+^ levels in cancer cells treated with Ler and Btz. Mechanistic studies employing various Ca^2+^ antagonists revealed that an MCU-mediated mitochondrial Ca^2+^ overload may be critical for Ler/Btz-induced mitochondrial dilation, subsequent ER dilation, and cell death. In contrast, an increase in cytosolic Ca^2+^ contributes solely to ER dilation at the later phase of Ler/Btz treatment. As the possible underlying mechanism, the authors proposed that the Ler/Btz-mediated stabilization of DHP receptor and RyR may result in the release of Ca^2+^ from the ER and the subsequent MCU-mediated mitochondrial Ca^2+^ uptake. This sustained mitochondrial Ca^2+^ overload may cause mitochondrial dilation and prompt opening of mPTP, leading to the leakage of Ca^2+^ into the cytosol (Lee et al., 2019).

### Loperamide + Proteasome Inhibitor

Kim et al. (2019) showed that the widely used anti-diarrheal drug, loperamide (Lop), effectively enhances the cytotoxicity of PIs in various colon cancer cells, but not in normal colon epithelial cells (Kim et al., 2019). The combination of sublethal concentrations of Btz and Lop (Lop/Btz) effectively triggered paraptosis-like cell death accompanied by severe vacuolation derived from the ER in various colon cancer cells, whereas either agent alone failed to induce notable vacuolation or cell death. In Lop/Btz-treated cancer cells, mitochondrial fragmentation was observed. Lop enhances Btz-mediated ER stress and ER dilation due to misfolded protein accumulation, leading to the upregulation of C/EBP homologous protein (CHOP) and subsequent paraptosis-like cell death. In addition, Btz/Lop increased both cytosolic and mitochondrial Ca^2+^ levels. An increase in Ca^2+^ (possibly by an influx of extracellular Ca^2+^) appears to play a critical role in the anticancer effects of Lop/Btz by affecting ER-derived vacuolation. Mitochondrial Ca^2+^ overload also contributes to Lop/Btz-mediated cytotoxicity, although it does not affect the dilations of the ER and the mitochondria (Kim et al., 2019).

### Nutlin-3 + Proteasome Inhibitor

The small molecule mouse double minute 2 homolog antagonist, Nutlin-3, exhibits promising therapeutic anti-cancer activity (Vassilev et al., 2004; Vassilev, 2007). Lee et al. (2017) showed that the combined treatment with Nutlin-3 and Btz triggered the formation of megamitochondria and progressive fusion of swollen ER, leading to paraptotic cell death in various p53-defective Btz-resistant solid tumor cells (Lee et al., 2017). Neither Nutlin-3 alone nor Btz alone did significantly affect the cellular morphology and viability, although Nutlin-3 alone induced a transient mitochondrial dilation. Mechanistically, proteasomal-impairment-induced ER stress (particularly CHOP upregulation) critically contributes to Nutlin-3/Btz-induced ER dilation and subsequent cell death induced by Nutlin-3/Btz. In addition, Nutlin-3/Btz, but not either agent alone, increased both cytosolic and mitochondrial Ca^2+^ levels. An increase in cytosolic Ca^2+^, possibly by an influx of extracellular Ca^2+^, plays an important role in Nutlin-3/Btz-induced ER dilation and subsequent cell death. For Nutlin-3/Btz-induced mitochondrial dilation, Nutlin-3-mediated mitochondrial stress due to the accumulation of misfolded proteins within the mitochondria may be more important than mitochondrial Ca^2+^ overload (Lee et al., 2017).

## Involvement of Ca^2+^ in Paraptosis

### Role of Ca^2+^ in Mitochondrial Dilation

Mitochondrial dilation is a key morphological feature of paraptosis, together with ER dilation (Sperandio et al., 2000). Mitochondrial dilation generally includes both mitochondrial swelling and their fusion to form megamitochondria (giant mitochondria) in paraptosis (Yoon et al., 2010, 2012, 2014; Yumnam et al., 2014; Lee et al., 2017; Fontana et al., 2019; Zhao et al., 2019). Mitochondrial swelling is defined as an increase in mitochondrial volume due to an influx of fluid and is known to occur first by expansion of the intracristal space and later by swelling of the matrix compartment (Trump and Ginn, 1968). Some degree of mitochondrial swelling may occur as a reversible, pre-lethal form of cellular damage (Halestrap, 1989; Lim et al., 2002). The mitochondria become enlarged at most three times, by simple swelling, compared to their original size (typically 0.5–1 μm in length). The size of the dilated mitochondria, including megamitochondria (with width and length > 1 μm) (Lee et al., 2017; Wieczorek et al., 2017; Palma et al., 2019), in paraptosis often exceed those of swollen mitochondria observed during apoptosis or necrosis. Swollen mitochondria can be distinguished from megamitochondria by other morphological features, in addition to the degree of enlargement. Swollen mitochondria are seen to be round with pale matrix and cristae are observed only on the periphery, whereas the shape of megamitochondria in early stages are often irregular and the density of their matrix appears well maintained (Teranishi et al., 1999). When megamitochondria are further swollen, they are seen with a pale matrix and their cristae are detected only on the periphery. Previously, a treatment with Ca-SANDOZ reportedly induce the formation of megamitochondria in brown adipocyte of Wistar rats (Golic et al., 2014), but the exact role and the regulatory mechanism of megamitochondria formation in paraptosis remain to be clarified. In the paraptosis induced by hesperidin (Yumnam et al., 2016), δ-TT (Fontana et al., 2020a), IPH4 (Yokoi et al., 2020), and iturin A-like lipopeptides (Zhao et al., 2019), the mitochondria undergo swelling and show cristae disintegration and loss. On the other hand, megamitochondria formation due to the fusion of mitochondria is observed in the paraptosis induced by curcumin (Yoon et al., 2010), celastrol (Yoon et al., 2014), CGP-37157/Btz (Yoon et al., 2012), and Nutlin-3/Btz (Lee et al., 2017).

Ca^2+^ and K^+^ play important roles in the physiological and pathological swelling of the mitochondria. Imbalances of these ions between the cytosol and matrix increase the osmotic pressure and enhance the water influx, leading to matrix swelling (Kaasik et al., 2007; Javadov et al., 2018). The transport of Ca^2+^ and K^+^ across the IMM is associated with influx/efflux mechanisms for other ions, such as Na^+^, Cl^–^, and H^+^ (Halestrap et al., 1986; Halestrap, 1994; Kaasik et al., 2007; Javadov et al., 2018). This complex interplay between mitochondrial swelling and ion homeostasis maintains the mitochondrial function and metabolism under physiological conditions. Mild Ca^2+^-mediated increases in matrix volume over the physiological range can promote oxidative phosphorylation and electron transfer chain and thus help satisfy the metabolic requirements of the cell (Halestrap, 1987, 1989; Lim et al., 2002). In addition, changes in IMM fluidity due to the altered mitochondrial shape and membrane tension may affect the activity of ion channels and other transporters, including the mechanosensitive mitochondrial large-conductance Ca^2+^-activated K^+^ channel (Walewska et al., 2018). Therefore, a slight increase of the matrix volume can regulate mitochondrial function and metabolism, possibly representing an adaptive response to protect the mitochondria against severe oxidative stress and delaying the onset of cell death. On the other hand, massive Ca^2+^ release from the ER causes the prolonged mitochondrial Ca^2+^ overload and excessive mitochondrial swelling (Rovere et al., 2016; Marchi et al., 2018). Mitochondrial Ca^2+^ overload has been commonly reported in paraptosis-associated cell death by curcumin (Yoon et al., 2012), celastrol (Yoon et al., 2014), hesperidin (Yumnam et al., 2016), morusin (Xue et al., 2018), δ-TT (Fontana et al., 2020a), IPH4 (Yokoi et al., 2020), Me_2_NNMe_2_ (Hager et al., 2018), CGP-37157/PI (Yoon et al., 2012), Ler/PI (Lee et al., 2019), Lop/PI (Kim et al., 2019), and Nutlin-3/PI (Lee et al., 2017). Among them, the functional significance of MCU-mediated mitochondrial Ca^2+^ overload as an early signal for paraptosis was confirmed by the reports that pharmacological and/or genetic inhibition of MCU attenuates the mitochondrial dilation and subsequent paraptotic cascades (i.e., ER stress, ER dilation, and subsequent cell death) induced by curcumin (Yoon et al., 2012), celastrol (Yoon et al., 2014), hesperidin (Yumnam et al., 2016), CGP-37157/PI (Yoon et al., 2012), and Ler/PI (Lee et al., 2019). VDAC-mediated mitochondria Ca^2+^ uptake was shown to be involved in morusin- or δ-TT-induced paraptotic cell death (Xue et al., 2018; Fontana et al., 2020a). The source of overloaded Ca^2+^ in the mitochondria was the ER (Yoon et al., 2012, 2014; Lee et al., 2019); RyR is reportedly involved in the release of Ca^2+^ from the ER in curcumin- or Ler/PI-induced paraptosis (Yoon et al., 2012; Lee et al., 2019), whereas IP_3_R was involved in that process during celastrol-induced cell death (Yoon et al., 2014). In addition, both RyR and IP_3_R were reported to mediate the release of Ca^2+^ from the ER during hesperidin-induced paraptosis (Yumnam et al., 2016). Furthermore, inhibition of the mNCX-mediated Ca^2+^ efflux from the mitochondria can contribute to mitochondrial Ca^2+^ accumulation and subsequent paraptotic events (Yoon et al., 2012). The combination of PI and the mNCX inhibitor, CGP-37157, induces paraptosis accompanied by sustained mitochondrial Ca^2+^ overload, mitochondrial dilation, ER stress, and ER dilation (Yoon et al., 2012). Collectively, these results suggest that an excessive Ca^2+^ overload in the mitochondria not only triggers mitochondrial dilation but also contributes to subsequent paraptotic events including ER dilation.

### Role of Ca^2+^ in ER Dilation

Appropriate maintenance of the intraluminal homeostasis in the ER is needed to keep the cellular viability, and ER dilation is the most common morphological feature of paraptosis (Yoon et al., 2010, 2012, 2014; Yumnam et al., 2014; Lee et al., 2017, 2019; Hager et al., 2018; Xue et al., 2018; Fontana et al., 2019; Kim et al., 2019). Regarding the involvement of Ca^2+^ in paraptosis, Ca^2+^ depletion within the ER can contribute to ER dilation during paraptosis. Maintenance of sufficient Ca^2+^ levels within the ER is crucial for the function of components of the protein folding machinery, such as chaperones and folding enzymes; this is because the chaperone activities of the molecular chaperones, Ig binding protein (Bip)/glucose-regulated protein 78 (GRP78), PDI, GRP94, and ERp57/PDIA3, are regulated by the binding of Ca^2+^ (Prins and Michalak, 2011; Carreras-Sureda et al., 2018). Therefore, alteration of the Ca^2+^ concentration within the ER by activation of IP_3_R or RyR can contribute to ER stress and ER dilation by causing incorrect protein folding and an accumulation of misfolded proteins. Mimnaugh et al. (2006) showed that geldanamycin/Btz induces ER-derived vacuolation and proposed that this effect was due to a massive accumulation of misfolded proteins within the ER lumen. The authors believed that this would exert an osmotic force, resulting in the induction of an influx of water from the cytoplasm and the distension of the ER into vacuoles. The increased ER volume provided by ER-derived vacuolation may reduce the amount of misfolded proteins in the ER and facilitate their utilization, providing cells with an adaptive cellular response under mild stress (Mimnaugh et al., 2006). However, long-term unresolved ER stress may trigger extensive fusion among the swollen ER membranes and subsequently irreversible ER-derived vacuolation, resulting in an ER-associated degradation-compromised condition and cell death (Shubin et al., 2016). Recent studies have shown that impairment of protein thiol homeostasis also plays a critical role in plumbagin-induced paraptosis-associated cell death (Binoy et al., 2019), likewise with gambogic acid (Seo et al., 2019), ophiobolin A (Kim et al., 2017), and PDTC/doxorubicin (Park et al., 2018). These paraptosis-inducing agents commonly harbor highly electrophilic features that are used to covalently modify free sulfhydryl or hydroxyl groups on proteins, causing protein misfolding, misfolded protein accumulation in the ER lumen, ER stress, and ER dilation (Kim et al., 2017; Park et al., 2018; Seo et al., 2019). The thiol-reactivity of these agents may be responsible for their inhibitory effects on proteasomal activity; in fact, most paraptosis-inducing agents demonstrate proteasome inhibitory activity (Yoon et al., 2010, 2014; Wang et al., 2012). In addition, the copper complex of Me_2_NNM_2_-mediated PDI inhibition was shown to be responsible for the induction of paraptosis by the disruption of the ER protein thiol homeostasis (Hager et al., 2018). The authors proposed that disruption of the ER thiol redox homeostasis by PDI inhibition contributes to Ca^2+^ release from the ER, organelle swelling, mitochondrial membrane depolarization, and subsequent cell death. Collectively, these findings suggest that the cross-modulation between imbalanced Ca^2+^ in the ER and impaired proteostasis may be part of the progression of paraptosis.

### Ca^2+^-Mediated Communication Between the ER and the Mitochondria in Paraptosis

**FIGURE 2:**
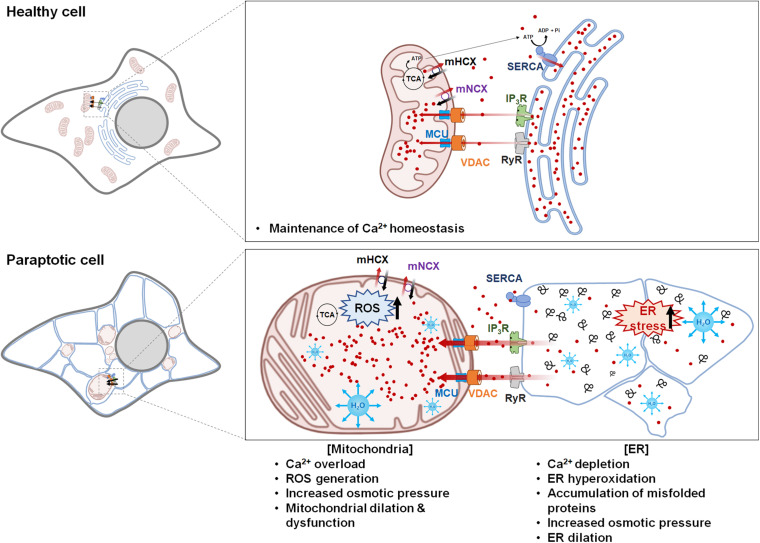
Hypothetical model for Ca^2+^-mediated communication between the mitochondria and the endoplasmic reticulum (ER) in paraptosis. Ca^2+^ is released from the ER through IP_3_Rs or RyRs and taken up into the mitochondria *via* voltage-dependent anion channel and mitochondrial Ca^2+^ uniporter. Mild mitochondrial Ca^2+^ uptake activates the tricarboxylic acid cycle and electron transfer chain, leading to ATP synthesis and reactive oxygen species (ROS) generation; this generates a positive feedback on IP_3_Rs and induces the further release of Ca^2+^ from the ER and its uptake into the mitochondria. However, prolonged Ca^2+^ overload in the mitochondria triggers mitochondrial membrane potential loss, mPTP opening, and the influx of water to the matrix, leading to mitochondrial swelling. The mitochondria cease to take up Ca^2+^ because there is no electrochemical gradient in the mitochondrial membrane, while the ER fails to take up Ca^2+^ because sarcoplasmic/ER Ca^2+^ ATPase is inhibited by ROS and ATP shortages. This increases the cytosolic Ca^2+^ level. In addition, excessive depletion of Ca^2+^ in the ER hampers the actions of chaperones, which depend on Ca^2+^ binding to promote protein folding in the ER; this contributes to the accumulation of misfolded proteins, leading to the water-influx-based distention of the ER lumen. This Ca^2+^-mediated vicious cycle between the ER and the mitochondria further expands mitochondria- and ER-derived vacuoles, leading to irreversible structural and functional defects in these two organelles and eventual cell death. The figure was produced using Biorender (biorender.com).

During the progression of paraptosis, the dilation of the mitochondria and the ER appears to be interdependently controlled, although mitochondrial dilation generally precedes ER dilation. Based on reports indicating that intracellular Ca^2+^ transport is involved in the paraptosis induced by various triggers, the following hypothetical model of the mechanisms underlying Ca^2+^-mediated paraptosis can be proposed (Figure 2).

The regulation of Ca^2+^ signaling by the ER depends on SERCA-mediated Ca^2+^ uptake activity (Vandecaetsbeek et al., 2011), the expression of luminal Ca^2+^ binding proteins, and the activity of Ca^2+^ release channels, including IP_3_R and RyR (Marks, 1997; Rovere et al., 2016). As an initial step of Ca^2+^-mediated paraptosis, Ca^2+^ is released from the ER to the MAM through IP_3_Rs or RyRs. The subsequent uptake of Ca^2+^ by the mitochondria is driven by the difference of membrane potential generated by the respiratory chain, which provides the electrochemical force needed for positively charged ions, such as Ca^2+^, to enter the mitochondrial matrix (Giorgi et al., 2018). VDAC at the OMM (Gincel et al., 2001; Rapizzi et al., 2002) and MCU at the IMM mediate the uptake of Ca^2+^ into the mitochondria (Baughman et al., 2011; De Stefani et al., 2011). Mild mitochondrial Ca^2+^ uptake can activate the tricarboxylic acid cycle and electron transport chain, triggering mitochondrial ROS generation and the release of H_2_O_2_ at the MAM (Feissner et al., 2009; Rizzuto et al., 2012). This may provide positive feedback to IP_3_Rs, enhancing their ability to sustain the release of Ca^2+^ from the ER and promote mitochondrial Ca^2+^ uptake. Furthermore, ER hyperoxidation due to thiol-oxidizing compounds also can promote Ca^2+^ release by opening of Ca^2+^ channels in the ER and inhibiting SERCA, thereby indirectly contributing to ER stress (Lizák et al., 2020). Interestingly, ROS generation was often associated with the paraptosis induced by Ca^2+^ homeostasis-perturbing drugs, including curcumin (Yoon et al., 2010), hesperidin (Yumnam et al., 2016), morusin (Xue et al., 2018), δ-TT (Fontana et al., 2020a), and chalcomoracin (Han et al., 2018), as shown in Table 1. However, the paraptosis induced by various drugs with thiol reactivity due to their high electrophilic nature, including Me_2_NNM_2_ (Hager et al., 2018), plumbagin (Binoy et al., 2019), gambogic acid (Seo et al., 2019), and ophiobolin A (Kim et al., 2017), was not correlated with ROS generation despite the fact that their vacuolation and cell death were successfully inhibited by the thiol-containing antioxidants. These results suggest that a thiol-dependent mechanism, rather than ROS, is critical for the anti-cancer activities of these drugs.

When considering the role of Ca^2+^ in mitochondrial dilation, it is notable that, at the early phase of paraptosis, Ca^2+^-mediated mitochondrial dilation may represent an adaptive response aimed at facilitating the removal of Ca^2+^ released from the ER and regulating the mitochondrial matrix volume by relieving stress to support the functional and morphological integrity of the mitochondria and delay the onset of cell death. The phenomenon of the formation of megamitochondria at the initial phase of paraptosis could be also considered as an adaptive process at the organelle level to unfavorable conditions, although further studies are definitely needed to prove this hypothesis. However, prolonged Ca^2+^ influx from the ER may exceed the loading capacity of the mitochondria; this could cause mitochondrial depolarization and compromise mitochondrial function, ultimately resulting in irreversible cell death. In support of this hypothesis, several studies have shown that mitochondrial Ca^2+^ overload triggers the production of ROS, which can further promote opening of mPTP (Hajnóczky et al., 2006; Görlach et al., 2015). This results in mitochondrial swelling because it allows water, ions, and other solutes to enter the matrix and equilibrate the solutes on both sides of the IMM (Kaasik et al., 2007; Javadov et al., 2018). Subsequent dissipation of the MMP results in uncoupling of oxidative phosphorylation and metabolic collapse. At the same time, loss of MMP might reduce the mitochondrial capacity to retain Ca^2+^, resulting in increased cytosolic Ca^2+^ levels. In addition, cytosolic Ca^2+^ cannot be taken up into the ER due to the shortage of ATP, which is required for the ability of SERCA to pump Ca^2+^ into the ER. As a result, Ca^2+^ is severely depleted in the ER; this leads to the accumulation of misfolded proteins in the ER lumen, contributing to further ER dilation. Expansion of the luminal space of the ER and the subsequent fusion of swollen ER progress to the point that most of the cellular space is occupied by large ER-derived vacuoles. Thus, sequential and multifactorial cross-modulation among mitochondrial Ca^2+^, ROS, and ΔΔ(Ψm may contribute to the sequential and cooperative dilation of the mitochondria and the ER in paraptosis.

## Involvement of Other Ions in Paraptosis

In addition to the disruption of Ca^2+^ homeostasis, imbalances of other ions, including K^+^ and Cl^–^, are reportedly involved in paraptosis. Previously, Hoa et al. (2007) reported that human monocytes/macrophages induce paraptotic cell death in human glioma cells expressing macrophage colony-stimulating factor; this occurs *via* activation of BKCa channels, which are found at the ER and the mitochondria. The authors hypothesized that, when BKCa channels open, K^+^ is expelled in exchange for the entry of Na^+^ to maintain the electroneutrality of the cell. The entry of Na^+^ results in the uptake of water, generating the observed cellular swelling and vacuolation (Hoa et al., 2007). Conversely, however, ophiobolin A was shown to induce paraptosis-like cell death in human glioblastoma cells by inhibiting BKCa channel activity (Bury et al., 2013). These authors proposed that BKCa inactivation would presumably induce the accumulation of K^+^ in the ER and the mitochondria, leading to the increased osmotic pressure responsible for vacuolation. Subsequently, opening of Ca^2+^ channels present in the membrane or the ER may also contribute to the paraptotic cascade. Future work will be needed to investigate how both the activation of BKCa and its inhibition can induce paraptosis. Furthermore, an increase in intracellular Cl^–^ concentration by the activation of chloride intracellular channel-1 (CLIC1) was shown to be involved in the paraptosis induced by a purified resin glycoside fraction (RFP) of Pharbitidis Semen (Zhu et al., 2019). Blockade of CLIC1 by DIDS (an inhibitor of anion channels) attenuated ER stress, cytoplasmic vacuolization, and cell death, suggesting that CLIC1 may act as a critical and early signal in RFP-induced paraptosis. Taken together, these results indicate that the movements of both cations and anions can contribute to the induction of paraptosis.

## Discussion

The inherent or acquired resistance of tumor cells to apoptosis is a major hurdle limiting successful anticancer therapy and the cause of many cancer-related deaths (Holohan et al., 2013). Therefore, targeting cancer cells by the induction of an alternative caspase-independent cell death pathway (Mathiasen and Jäättelä, 2002), such as paraptosis, offers the opportunity to overcome apoptosis resistance in cancer cells. Rapidly proliferating and aneuploid cancer cells demand a much higher level of protein-folding activity in the ER, compared to normal cells (Deshaies, 2014). Additionally, cancer cells exhibit greater oxidative stress than normal cells due (at least partially) to oncogenic activation and altered mitochondrial activity (Gupta et al., 2011). Since paraptosis-inducing agents simultaneously target both the mitochondria and the ER, which are already under stress in cancer cells, therapeutic strategies aimed at inducing paraptosis may offer a two-pronged attack strategy to selectively kill cancer cells.

Although various models have been proposed to classify programmed cell death, exclusive definitions are difficult to conceptualize and probably factitious, given the crosstalk between cell death signaling pathway (Bröker et al., 2005). In addition, some morphological and biochemical features are shared in different cell death modes. Excessive swelling due to prolonged mitochondrial Ca^2+^ overload was reported to be able to induce apoptosis or necrosis, depending on the availability of ATP (Rovere et al., 2016; Marchi et al., 2018). In apoptosis, mitochondrial outer membrane permeabilization is critical for the release of cytochrome c and other molecules, leading to the activation of caspases (Green and Kroemer, 1998). Although mitochondrial swelling is also an important morphological feature of paraptosis, no release of mitochondrial cytochrome c (Valamanesh et al., 2007; Gandin et al., 2012; Yoon et al., 2014) and/or AIF (Valamanesh et al., 2007) into the cytosol was shown in several models of paraptosis. In addition, no requirement of caspases has been reported in many studies on paraptosis (Lee et al., 2016; Fontana et al., 2020b). Mitochondrial swelling is also a key feature of oncosis, the cell death mode accompanied by cellular swelling, organelle swelling, blebbing, and increased membrane permeability, resulting in bursting of the plasma membrane (Majno and Joris, 1995; Weerasinghe and Buja, 2012). However, paraptosis is not associated with cell swelling. Furthermore, obvious damage to the OMM has not been shown in transmission electron microscopy pictures of cells undergoing paraptosis (Yoon et al., 2010, 2014; He et al., 2018; Xue et al., 2018; Yokoi et al., 2020). MMP was fairly maintained in the progression of paraptosis, but its loss was detected at the excessively expanded megamitochondria at the late stage of paraptosis (Lee et al., 2019; Seo et al., 2019). Reduced ATP synthesis is also observed following the extensive vacuolation derived from the mitochondria and/or the ER in paraptosis (He et al., 2018; Binoy et al., 2019; Nedungadi et al., 2019). Therefore, we presume that swelling and fusion of the mitochondria at the pre-lethal phase may provide an adaptive response that helps maintain mitochondrial membrane integrity and function before leading to irreversible paraptotic cell death. Further studies on the detailed changes in the structure/function of the mitochondria as well as the relationship between Ca^2+^ imbalance and the integrity of mitochondrial membrane during the progression of paraptosis will be helpful to clearly understand paraptosis and clarify the differences among various cell death modes accompanied by mitochondrial Ca^2+^ overload and swelling.

While curcumin (Yoon et al., 2010), celastrol (Yoon et al., 2014), hesperidin (Yumnam et al., 2014), IPH4 (Yokoi et al., 2020), and Me_2_NNMe_2_ (Hager et al., 2018) induce paraptosis independently of apoptosis and/or autophagy in the tested cancer cells, several agents, including δ-tocotrienol (Fontana et al., 2019, 2020a), chalcomorain (Han et al., 2018), and iturin A-like lipopeptides (Zhao et al., 2019), induce not only paraptosis but also simultaneous apoptosis and/or autophagy, as shown in Table 1. It is intriguing to speculate why the same agent can induce different cellular fates in some types of cells. The cellular response to a cytotoxic drug can be affected by various factors, including the type and dose of the drug, the extent of cytotoxic stress exerted to the cells, and the cellular context. It has been shown that more than one kind of cell death program can be activated at the same time (Unal-Cevik et al., 2004). The dominant cell death phenotype may be determined by the relative speed and effectiveness of the available death programs: In response to cellular damage, the characteristics of several death pathways can be exhibited, but in general, only the most effective and the fastest pathway is evident (Bröker et al., 2005). Furthermore, cellular heterogeneity (even within the same cell line) can contribute to the ability of a given drug to induce different cell death modes (Inde and Dixon, 2018). We now know that many cancer cells are not homogeneous in nature but rather contain a variety of cells with different characteristics (Meacham and Morrison, 2013; Dagogo-Jack and Shaw, 2018). Thus, the presence of heterogeneous populations of cancer cells may yield different drug sensitivities, treatment resistances, and the induction of different or mixed types of cell death modes.

Although the molecular basis of paraptosis needs further investigation, many reports have indicated that impaired proteostasis is critical for the induction of paraptosis, as evidenced by the accumulation of poly-ubiquitinated proteins and ER stress marker proteins as well as the ability of the protein synthesis blocker to effectively inhibit vacuolation and cell death (Lee et al., 2016; Fontana et al., 2020b). As shown in Table 1, various chemicals have been shown to induce Ca^2+^ imbalance-mediated paraptosis. Interestingly, these Ca^2+^ homeostasis-disrupting drugs also induced proteostatic impairment, leading to the accumulation of poly-ubiquitinated proteins and ER stress marker proteins. Genetic or pharmacological inhibition of Ca^2+^ imbalance effectively blocked this proteostatic impairment and subsequent paraptosis (Yoon et al., 2010, 2014). These results suggest that disruption of Ca^2+^ homeostasis may negatively affect cellular proteostasis and enhance proteotoxicity, contributing to paraptosis. Pretreatment with CHX almost completely inhibited Ca^2+^ release from the ER, mitochondrial Ca^2+^ overload, disruption of proteostasis, and subsequent paraptotic events (Yoon et al., 2010, 2012; Yumnam et al., 2014; Lee et al., 2019). Taken together, these results suggest that Ca^2+^ imbalance and proteostatic disruption are interdependently regulated during the progression of paraptosis. In addition, simultaneous disruption of Ca^2+^ homeostasis and proteostasis, rather than induction of Ca^2+^ imbalance or proteostatic disruption alone, may be an effective means to kill cancer cells by inducing paraptosis (Yoon et al., 2012; Kim et al., 2019; Lee et al., 2019).

Btz, the first PI approved by FDA for clinical application, has shown efficacy in both front-line and relapsed/refractory settings, but its use can be limited by the development of resistance and/or side effects. The principal dose-limiting toxic effects of Btz are peripheral neuropathy, thrombocytopenia, neutropenia, fatigue, and diarrhea (Dou and Zonder, 2014). In addition, the PIs have shown limited clinical efficacy as monotherapies for solid tumors (Huang et al., 2014). However, Ca^2+^ homeostasis-perturbing drugs, such as lercanidipine, loperamide, and CGP-37157, act together with PIs to induce paraptosis (Yoon et al., 2012; Kim et al., 2019; Lee et al., 2019). Treatment with these Ca^2+^ homeostasis-perturbing drugs alone slightly and transiently induced mitochondrial dilation without dilation of the ER, whereas treatment with the PIs alone was not enough to induce paraptosis in these studies. It is possible that either Ca^2+^ imbalance or proteasome inhibition may trigger cellular adaptive responses, including the autophagy, UPR, and reactivation of the ubiquitin–proteasome system, contributing to the restoration of cellular homeostasis and viability (Cooper et al., 1997; Brostrom and Brostrom, 2003; Ding et al., 2007). Additionally, the proteasome is involved in the degradation of both IP_3_R (Oberdorf et al., 1999) and RyR (Pedrozo et al., 2010); thus, the PI-mediated stabilization of these Ca^2+^ channels may induce the enhanced and sustained release of Ca^2+^ from the ER in cells treated with these Ca^2+^ homeostasis-perturbing drugs, contributing to the effective induction of paraptosis. A combination of Ca^2+^ homeostasis-perturbing drugs and PIs may therefore effectively induce paraptosis by triggering a vicious cycle of ER–mitochondria Ca^2+^ signaling. Furthermore, the combined regimen of a PI plus Ca^2+^-modulating clinical drugs may provide us many advantages, including enhanced therapeutic effectiveness, better safety due to the reduced side effects of a PI by the use of its lower dose, and the potential to expand the applicability of PIs to solid tumors (Kim et al., 2019; Lee et al., 2019). Therefore, induction of paraptosis through modulation of Ca^2+^ signaling may provide a novel therapeutic strategy to overcome the resistance of solid tumors against PIs in the future. It will be intriguing to explore the processes/regulatory pathways underlying the differences between ER and mitochondrial Ca^2+^ transfer in cancer cells vs. normal cells under paraptosis-inducing conditions. Further investigation of the regulatory mechanisms of Ca^2+^ in paraptosis may provide new insights into paraptosis and potential therapeutic strategies for the treatment of resistant cancer.

## Author Contributions

EK and KSC wrote the manuscript. KSC supervised the preparation of the manuscript. DML created the figures. MJS and HJL created the table. All the authors contributed to the article and approved the submitted version.

## Conflict of Interest

The authors declare that the research was conducted in the absence of any commercial or financial relationships that could be construed as a potential conflict of interest.
